# The interaction between dendritic cells and T follicular helper cells drives inflammatory bowel disease: a review

**DOI:** 10.3389/fimmu.2026.1725349

**Published:** 2026-02-03

**Authors:** You Lv, Yan-Ling Jin, Ze Zhou, Jia-Bao Liao, Zhu-Quan Zhang, Lin-Yu Tang, Xue-Hua Xie, Si Wang, Meng-Xue Jin, Hong-Yi Liu

**Affiliations:** 1 College of Basic Medical Sciences, Yunnan University of Chinese Medicine, Kunming, Yunnan, China; 2 The First Clinical School of Medicine, Yunnan University of Chinese Medicine, Kunming, Yunnan, China; 3 The Second Affiliated Hospital of Yunnan University of Chinese Medicine, Kunming, Yunnan, China; 4 The Third Clinical Medical College of Yunnan University of Chinese Medicine, Kunming, Yunnan, China

**Keywords:** cell–cell interactions, dendritic cells, immunology, inflammatory bowel disease, T follicular helper cells

## Abstract

Inflammatory bowel disease (IBD), encompassing Crohn’s disease (CD) and ulcerative colitis (UC), has an important pathogenesis that lies in the self-amplifying inflammatory circuit formed by bidirectional interactions between dendritic cells (DCs) and T follicular helper (TFH) cells. This review elucidates that specific mature DC subsets in the intestinal inflammatory microenvironment drive TFH cell differentiation through synergistic co-stimulatory signals (CD80/CD86-CD28, OX40L-OX40) and cytokine networks (IL-12/STAT4/BCL-6, TGF-β/c-Maf/CXCR5); conversely, TFH-derived Lymphotoxin alpha 1 beta 2 (LTα1β2) activates stromal cell LTβR/NF-κB signaling pathway, inducing chemokine (CXCL13, CCL19, CCL21) production, thereby recruiting CCR7^+^ DC and CXCR5^+^ lymphocytes to form structural lymphoid clusters. Within these clusters, sustained DC-TFH cell interactions enhance TFH pathological effector functions (e.g., excessive IL-21 secretion), promote Th1/Th17 differentiation and weaken regulatory T cell inhibitory capacity, ultimately causing barrier destruction and tissue damage. Notably, while this pathogenic axis is active in both CD and UC, its cellular dynamics and microenvironment may exhibit disease-subtype distinctions. Current therapeutic strategies targeting this axis—including JAK inhibitors (e.g., upadacitinib), cytokine biologics (e.g., ustekinumab) and integrin blockers (e.g., vedolizumab)—achieve efficacy by interfering with DC-dependent TFH differentiation or TFH-mediated DC aggregation. Emerging evidence indicates traditional Chinese medicine active components (e.g., ginsenoside Rh2, curcumin) may intervene in this interaction through multi-pathway immunoregulation. However, utilizing single-cell and spatial transcriptomics to analyze spatial characteristics and disease-subtype-specific profiles of DC-TFH cell interactions remains key to developing next-generation therapies. While this axis provides a novel perspective for understanding immune dysregulation in IBD, its temporal role in disease initiation, crosstalk with other immune pathways, and translation from animal models to human disease remain challenges and future directions for the field.

## 1 Introduction

Inflammatory bowel disease (IBD) is a non-infectious, chronic, gastrointestinal inflammatory disorder characterized by alternating periods of relapse and remission, heterogeneous clinical manifestations, and the presence of one or more extraintestinal manifestations (1). IBD mainly includes two subtypes—Crohn’s disease (CD) and ulcerative colitis (UC) (2)—While both diseases predominantly affect the gastrointestinal tract, they differ in the specific sites and extent of involvement. For instance, CD can involve any part of the gastrointestinal tract from the mouth to the anus, while UC is mostly confined to the colon and rectum (3, 4). Beyond anatomical distribution, CD and UC are also characterized by distinct immunopathological features: CD is typically associated with a T helper 1 (Th1)/Th17-driven transmural inflammation, whereas UC often involves a more superficial, mucosal inflammation with a prominent Th2 component. Recurrent episodes of the disease can lead to complications such as perforation, fistula, toxic megacolon, and cancer, which can greatly affect the professional and personal life of patients. A meta-analysis showed that the incidence of colon cancer progressively increases with disease duration, rising from 1.6% at 10 years to 8.3% at 20 years, and further to 18.4% at 30 years (5), thereby imposing a substantial global public health burden.

Although traditional anti-IBD drugs such as 5-aminosalicylic acid, azathioprine, methotrexate, and corticosteroids have proven effective in the treatment of the disease, the acceleration of globalization and changes in dietary patterns have inevitably led to a rapid increase in the global prevalence of IBD. It is estimated that over 6.8 million people are affected by this condition worldwide. The geographical pattern of IBD incidence has gradually expanded from an initial predominance in Western countries to encompass developing countries in Asia, Eastern Europe, and Africa. For instance, in India, the incidence rate of IBD is reported at 9.31 per 100, 000 person-years (6). The main reason for the spread of IBD is the incomplete understanding of its driving factors. However, it is well established that the onset of IBD results from complex interactions between genetic and environmental factors, including diet, with gut dysbiosis, intestinal barrier damage, and abnormal immune responses all making significant contributions (7, 8). Specifically, inflammation in IBD is initially triggered by the innate immune response, primarily in response to intestinal barrier damage. Gut dysbiosis has been confirmed to be a key link in amplifying and maintaining the intestinal inflammatory response, further exacerbating intestinal barrier disruption (9–11) This process provides co-stimulatory signals for subsequent adaptive immune activation, ultimately promoting disease progression (11). Consequently, a comprehensive investigation into immune cell-targeting strategies holds promise for the dynamic monitoring of IBD as well as for improving disease outcomes (11–14).

Over recent years, the dynamic interaction between dendritic cells (DCs) and T follicular helper (TFH) cells has received increasing attention for its central role in driving IBD pathogenesis. This interaction constitutes a key pro-inflammatory positive feedback loop: in mouse mesenteric lymph node studies, specific mature DC subsets can effectively induce the differentiation of pro-inflammatory, dysfunctional TFH cells (15). Notably, this DC-TFH axis is active in both CD and UC, though its cellular composition and microenvironmental triggers may differ between the two subtypes. Meanwhile, these abnormally activated TFH cells, which express C-X-C motif chemokine receptor 5 (CXCR5), promote the recruitment and local activation of specific DC subsets or their precursors to sites of inflammation. They do so by participating in the shaping of the microenvironment of gut-associated lymphoid tissues, such as germinal centers (GCs), partly by promoting the production of chemokines such as C-X-C motif chemokine 13 (CXCL13) (16). This bidirectional interaction leads to the establishment of a close DC-TFH cell interaction network in the follicle-associated regions of tertiary lymphoid structures formed in secondary lymphoid organs, such as mesenteric lymph nodes, or at the sites of intestinal inflammation.

Within this network, DCs continuously provide excessive antigen presentation and co-stimulatory signals, such as CD80/CD86 and inducible co-stimulator ligand (ICOSL). These not only maintain and expand the population of dysfunctional TFH cells but also significantly enhance their effector functions, including the high-level secretion of pro-inflammatory cytokines such as interleukin-21 (IL-21) (15, 17–19), and directly exacerbate local inflammatory responses. The inflammatory microenvironment maintained by DC-TFH cell interactions and the effector factors produced by TFH cells, especially IL-21, further drive the differentiation, activation, and expansion of various pro-inflammatory effector T-cell subsets (such as Th1 and Th17 cells) (20, 21). These activated effector T cells migrate to the intestinal lamina propria, where they directly mediate epithelial barrier disruption and intestinal tissue damage, which jointly promote IBD pathology.

Accordingly, in this review, we discuss the key roles of DCs and TFH cells in the occurrence and development of IBD and delve into the core pro-inflammatory mechanisms established.

## 2 The function of DCs is related to their state

**Figure 1:**
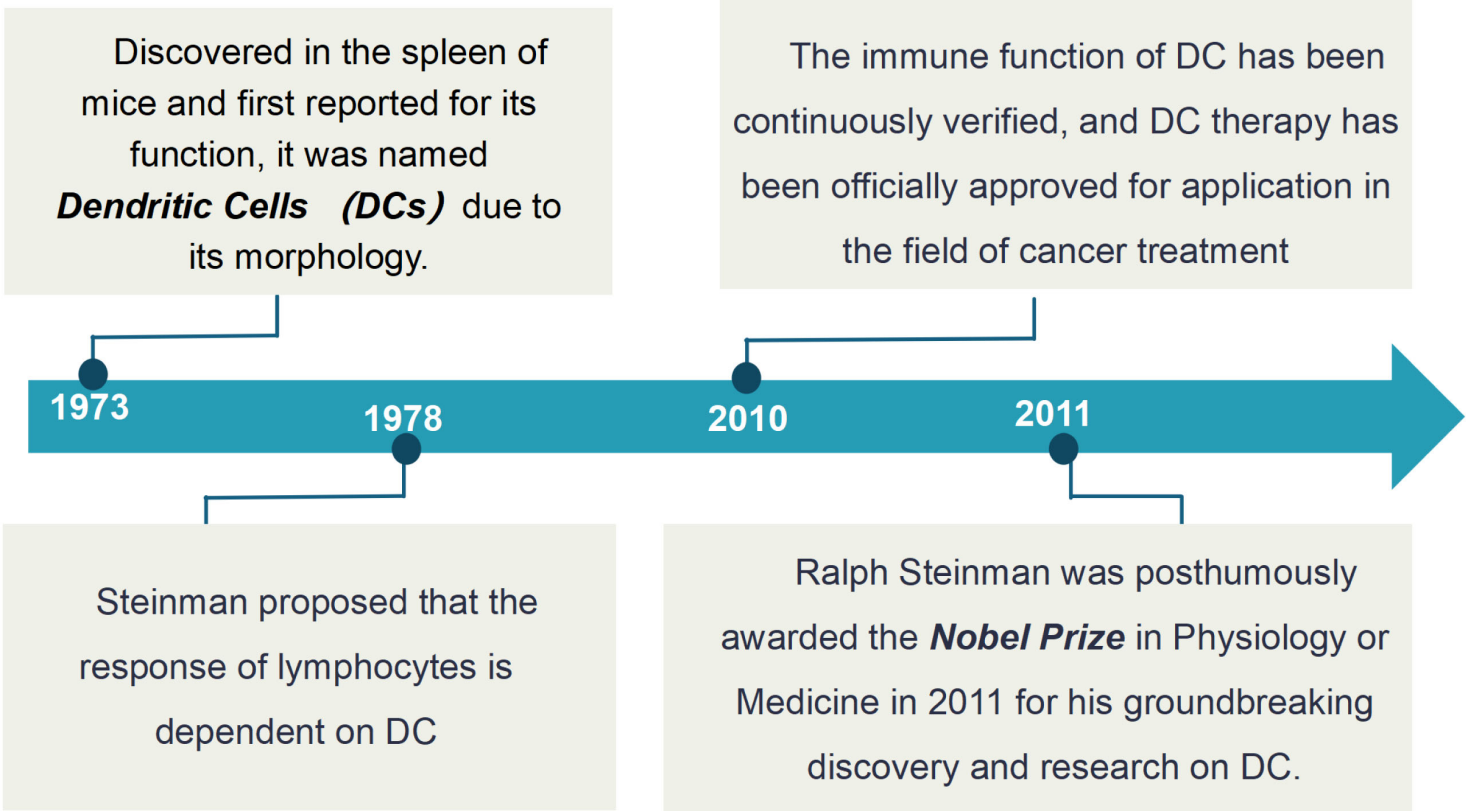
The historical development of DCs. The initial discovery of DCs, characterized by their stellate or dendritic protrusions, was made in the mouse spleen in 1973, with their phagocytic function reported simultaneously. In 1978, it was confirmed that the immune response of lymphocytes is mainly dependent on DCs. The discovery of DCs and their role in adaptive immunity was recognized with the *Nobel Prize* in 2011. DCs recognize, capture, and phagocytose pathogens, presenting antigenic information to T cells to activate adaptive immunity. In 2010, DC cell therapy was officially approved for cancer treatment.

As early as 1973, Ralph Steinman and Zanvil Cohn made the initial discovery of a rare type of cell in the mouse spleen. These cells had stellate or dendritic protrusions on their surface, from which DCs derive their name. Simultaneously, the phagocytic function of DCs was also reported for the first time (22), marking the formal introduction of DCs into the scientific research landscape. In 1978, Steinman’s group further confirmed that the immune response of lymphocytes is mainly dependent on DCs (23), and, in 2011, he was awarded the *Nobel Prize* in Physiology or Medicine for his contribution to the discovery of DCs and their role in adaptive immunity. Since then, an increasing number of studies have confirmed that DCs can recognize, capture, and phagocytose pathogens, breaking them down into small molecular fragments, which DCs then present on their cell surface. DCs then transport these fragments to lymphocyte-rich areas, where they present antigenic information to T cells, thereby activating adaptive immunity and directing them to effectively eliminate foreign invaders. Given these findings, in 2010, DC cell therapy was officially approved for cancer treatment, underscoring that the discovery of DCs not only changed the understanding of the mechanism underlying immune system function but also promoted the development of immunotherapy (24) (**Figure 1**).

**Figure 2:**
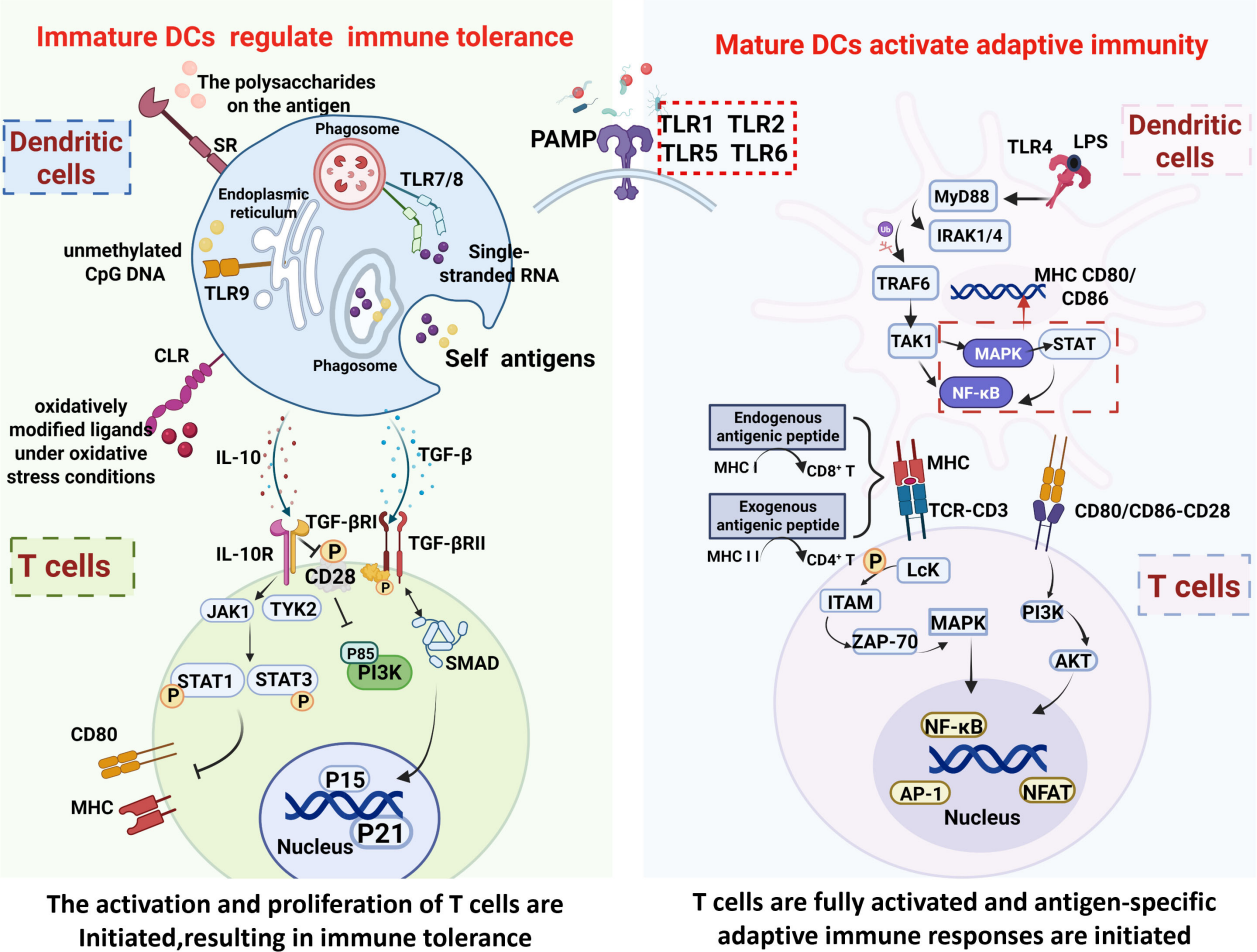
The functional transition of DCs from an immature to a mature state. Immature DCs act as “phagocytic cells” with immune-tolerant regulatory effects, internalizing antigens and inhibiting T-cell activity. Upon encountering pathogens, DCs mature, express high levels of co-stimulatory and MHC molecules, process antigens, and present them to T cells, thereby becoming “bridge cells” that activate adaptive immunity.

DCs exhibit marked heterogeneity and can be subdivided into distinct subsets according to their ontogeny, surface markers and functional specialisation. Each subset performs dedicated/specialized functions in intestinal immune surveillance and response. As illustrated in **Table 1**, the major DC compartments comprise conventional dendritic cells (cDCs, further separable into cDC1 and cDC2) (25), plasmacytoid dendritic cells (pDCs), monocyte-derived dendritic cells (Mo-DCs) and mature DCs enriched in immunoregulatory molecules (mregDC). Each subset displays a unique repertoire of surface receptors and performs dedicated tasks in antigen capture, processing, presentation and cytokine secretion, thereby tailoring specific immune responses in different tissue environments. The functional characteristics of the different subtypes are determined by their developmental pathway. However, the function of DCs is mainly related to their maturation state. Immature DCs do not possess antigen-presenting ability but can migrate to almost all lymphoid tissues throughout the body. They internalize antigens through phagocytosis and pinocytosis and exhibit an immune-tolerant phenotype toward substances recognized as “self” antigens (26). In this immature state, DCs play a role in inhibiting T-cell activity and contribute to the regulation of immune tolerance, acting as “phagocytic cells” with immune-tolerant regulatory effects. However, when they recognize “foreign” pathogens, DCs bind pathogen-associated molecular patterns (PAMPs) through pattern recognition receptors (PRRs) on their surface, and begin to differentiate and mature. They then express high levels of both co-stimulatory and MHC molecules. Meanwhile, they process and synthesize the ingested antigens into antigenic peptides, load them onto MHC molecules, migrate from peripheral tissues into adjacent secondary lymphoid tissues, and present the antigens on the MHC molecules to T cells. After specific binding, they activate adaptive immunity and become “bridge cells” linking innate and adaptive immunity (26, 27) (**Figure 2**).

**Table 1:** Major dendritic cell subsets in intestinal immunity.

Subset	Key surface markers	Main functional characteristics	Secretory profile	Role in intestinal immunity
cDC1	CD103^+^, XCR1^+^, CLEC9A^+^ (172)	Cross-presentation; priming of cytotoxic CD8^+^ T cells and Th1 responses (173).	IL-12, IFN-λ, CXCL9/1 (174–176)	Critical for anti-viral/bacterial defense; promotes type 1 immunity (177).
cDC2	CD11b^+^, SIRPα^+^, CD1c^+^ (172)	Presentation of antigens to CD4^+^ T cells; initiation of diverse helper T cell responses (178).	CCL17, CCL2 (179)	Key orchestrator of mucosal CD4^+^ T cell responses; involved in homeostasis and inflammation (180).
pDC	CD123^+^, BDCA-2^+^, TLR7/9^+^ (181)	Professional producer of type I interferons in response to nucleic acids (182).	IFN-α/β, TNF-α (183)	Immune tolerance; anti-infection defense (184).
Mo-DC	CD14^+^, CCR2^+^ (185)	Differentiates from blood monocytes during inflammation; highly phagocytic (186).	TNF-α, IL-6, IL-23 (187)	Amplifies inflammatory responses in active lesions; contributes to tissue damage (188).
mregDC	CCL22^+^, LAMP3^+^ (189)	Associated with immunoregulatory functions (190).	CCL22, CCR7 (191)	——

### 2.1 Immature DCs have phagocytic and immune tolerance regulatory functions

Under physiological conditions, DCs exist in a dynamic immature state (28). Immature DCs have a smooth surface and exhibit elevated expression of phagocytosis-related receptors, including Toll-like receptors (TLRs), C-type lectin receptors (CLRs), and scavenger receptors (SRs) (29–32). These receptors contribute to the recognition of “self” antigens such as nucleic acids (DNA/RNA) and histones released after necrosis or apoptosis. For example, TLR9 can recognize unmethylated CpG DNA exposed after cell damage (33), while TLR7 and TLR8 primarily recognize single-stranded RNA (33, 34). C-type lectin receptors mediate glycan recognition on antigens (35), and scavenger receptors often help recognize oxidatively modified ligands under oxidative stress conditions (36). Subsequently, these phagocytosis-related receptors bind to “self” antigens, forming phagocytic cups that continuously close, encapsulating and internalizing the antigens within membrane-bound vesicles called phagosomes. The phagosomes fuse with lysosomes to process the antigens, which are then loaded onto MHC molecules on the surface of DC cells (32). Moreover, as apoptotic cells lack the necessary stimulating signals to activate co-stimulatory molecules and MHC molecules on the surface of immature DCs, the initiation of T cell-mediated adaptive immunity is transiently impeded (37).

When processing “self” antigens, immature DCs concurrently guide T cells to respond to “self” antigens in an immunosuppressive manner by secreting immunosuppressive factors such as IL-10 (38) and transforming growth factor-beta (TGF-β). Thus, in addition to phagocytosis, the regulation of immune tolerance represents another key function of immature DCs (39–41). After binding to receptors on the surface of T cells, IL-10 can activate Janus kinase 1 (JAK1) and tyrosine kinase 2 (TYK2), leading to the phosphorylation of signal transducer and activator of transcription 1 (STAT1) and STAT3, thereby inhibiting the activation of Th1 cells and reducing the production of pro-inflammatory cytokines (42–44). Furthermore, under the regulation of IL-10, the expression of co-stimulatory molecule CD80 and MHC on the surface of DCs is significantly reduced (45, 46). Additionally, IL-10 inhibits tyrosine phosphorylation in the T cell-specific surface glycoprotein CD28 and prevents the binding of phosphatidylinositol 3-kinase (PI3K) p85, thereby blocking the CD28 signaling pathway (47). As a result, T-cell polarization is inhibited due to the lack of co-stimulatory signals. TGF-β mainly regulates the proliferation and activation of T cells by specifically binding to TGF-β receptor II (TGF-βRII), leading to the recruitment of TGF-β receptor I (TGF-βRI) and the formation of a signaling complex composed of TGF-β/TGF-βRI/TGF-βRII. Once this complex has formed, TGF-βRII, which possesses intrinsic kinase activity, phosphorylates and activates TGF-βRI, leading to its dissociation from the signaling inhibitor FK506-binding protein 1A (FKBP1A). Activated TGF-βRI subsequently interacts with receptor-regulated small mothers against decapentaplegic (SMAD) proteins (R-SMADs), which are presented to TGF-βRI, and then phosphorylated and activated under the mediation of the adaptor protein SARA. Phosphorylated R-SMADs subsequently combine with SMAD4, forming a heterotrimeric complex (48). This complex translocates to the nucleus where it binds to specific DNA sequences and interacts with various transcription factors and co-regulators. Through this transcriptional regulation, the SMAD complex upregulates genes encoding cell cycle inhibitors (e.g., p15, p21), which arrest T cell proliferation, and promotes the expression of genes involved in the differentiation and function of regulatory T cells (Tregs). TGF-β signaling also modulates T cell receptor signaling thresholds and inhibits pro-inflammatory cytokine production, collectively suppressing T cell activation and proliferation, and enforcing immune tolerance (49, 50).

### 2.2 Following stimulation by “foreign” pathogens, DCs differentiate and mature, and their antigen-presenting ability is enhanced

When immature DCs recognize PAMPs of “foreign” pathogens such as bacteria and viruses through their surface PRRs, they initiate a maturation and differentiation program and activate a downstream signaling network (51). For instance, once TLR4 specifically recognizes lipopolysaccharide (LPS) from Gram-negative bacteria, it recruits interleukin-1 receptor-associated kinase 1/4 (IRAK1/4) through the myeloid differentiation factor 88 (MyD88) adaptor protein. This complex, in turn, mediates the polymerization and ubiquitination of tumor necrosis factor receptor-associated factor 6 (TRAF6), ultimately leading to the activation of transforming growth factor β-activated kinase 1 (TAK1). Signaling then diverges into two key pathways, involving either the secretion of inflammatory cytokines through the nuclear factor-kappa B (NF-κB) pathway or the regulation of cell proliferation through the mitogen-activated protein kinase (MAPK) pathway (52, 53). Notably, NF-κB, a core regulator of DC maturation, can directly bind to the promoter regions of genes encoding MHC and co-stimulatory molecules such as CD80/CD86 (54, 55). The MAPK pathway, meanwhile, indirectly enhances the expression of the above-mentioned immune molecules by activating transcription factors such as NF-κB and STAT proteins (56, 57). Following DC maturation, the expression of MHC molecules on the surface of these cells is significantly upregulated. MHC class I molecules present endogenous antigenic peptides, such as those derived from viruses, to CD8^+^ T cells, whereas MHC class II molecules present exogenous antigenic peptides, such as degradation products of bacterial proteins, to CD4^+^ T cells, thereby initiating specific T-cell responses (58–60).

However, effective T-cell activation strictly follows the two-signal activation principle. In addition to the first signal mediated by the MHC-antigenic peptide-TCR complex, the second signal provided by co-stimulatory molecules, such as CD80/CD86 and CD40, on the surface of DCs is also crucial for the stability of the immunological synapse and downstream signal transduction. When the TCR-CD3 complex recognizes the MHC-antigenic peptide complex (the first signal), lymphocyte-specific tyrosine kinase (Lck) phosphorylates immunoreceptor tyrosine-based activation motifs (ITAMs), leading to the recruitment and activation of ζ-chain-associated protein kinase 70 (ZAP-70), and, ultimately, the activation of the MAPK signaling cascade (61–63). Meanwhile, the binding of the co-stimulatory molecules CD80 and CD86 on DCs to CD28 on T cells (the second signal) triggers the phosphorylation of tyrosine residues in the intracellular domain of CD28, leading to the recruitment of phosphatidylinositol 3-kinase (PI3K) via its SH2 domains and, eventually, the activation of the PI3K-AKT pathway. Ultimately, the two signals are precisely coordinated in both temporal and spatial dimensions: the antigen-specific recognition—the first signal—provides the basic framework for activation, while the second signal dynamically regulates metabolic reprogramming and cell cycle progression through the PI3K-AKT pathway. Finally, through the synergistic action of the nuclear factor of activated T-cells (NFAT)/activator protein 1 (AP-1) and NF-κB transcription complexes, T cells are fully activated and antigen-specific adaptive immune responses are initiated (64, 65).

## 3 Adaptive immune activation initiates TFH cell differentiation and pro-inflammatory functions

TFH cells were first described in human lymphoid tissues in 1998 as CXCR5^+^CD4^+^ T cells residing within B cell follicles, although their functional identity as a distinct T helper subset remained undefined for decades (66). A major conceptual advance came in 2000, when CXCR5^+^ T cells were shown to migrate into follicles and deliver essential help to B cells, distinguishing them from classical Th1 and Th2 lineages. This led to the formal recognition of TFH cells as a separate effector T cell population (67).

The molecular identity of TFH cells was refined in 2005, when microarray analyses revealed a transcriptional signature unique to this subset, underscoring their specialized role in humoral immunity. Subsequent studies identified Bcl6 as the master transcription factor governing TFH differentiation, solidifying their developmental independence from other CD4^+^ T helper subsets (68). In 2009, the RING-type ubiquitin ligase Roquin was implicated in restraining TFH responses by destabilizing ICOS mRNA; its deficiency resulted in TFH hyperaccumulation and systemic autoimmunity, emphasizing the importance of post-transcriptional regulation in immune tolerance (69, 70).

**Figure 3:**
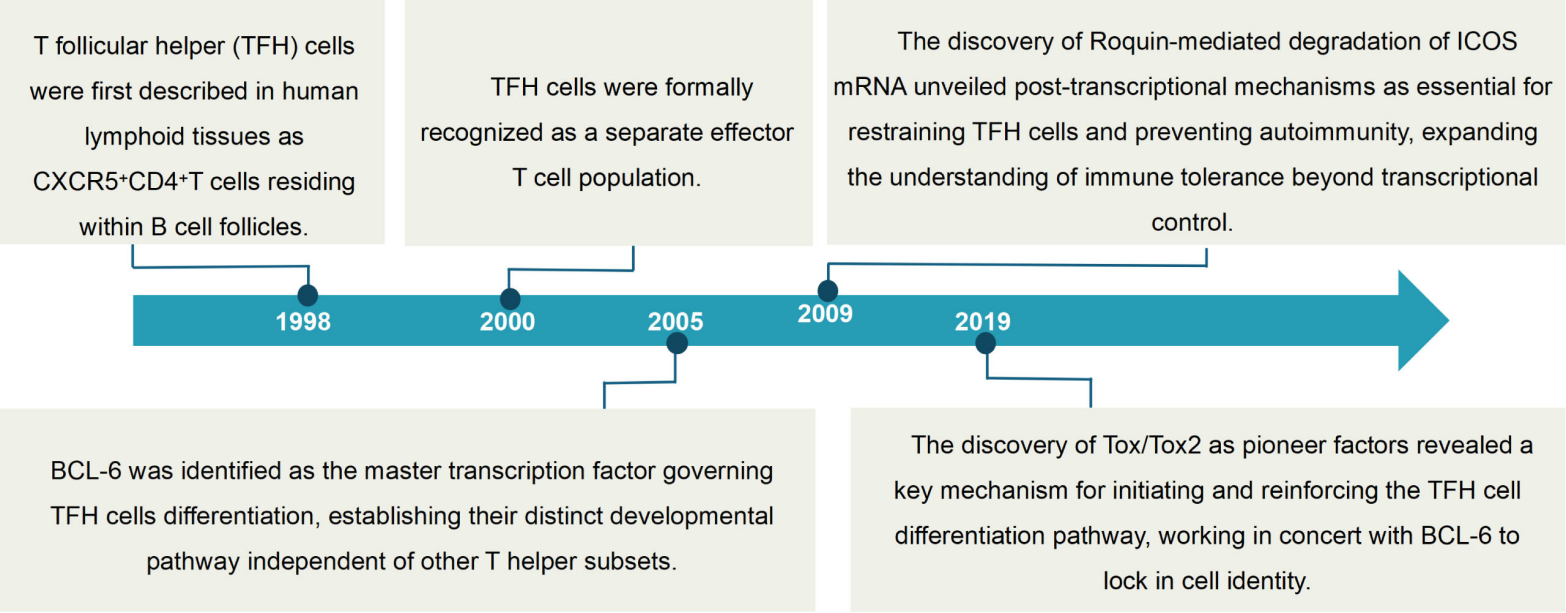
The historical development of TFH Cells. TFH cells were first described in human lymphoid tissues in 1998. Their functional identity was established in 2000 when CXCR5^+^ T cells were shown to migrate into follicles and deliver essential help to B cells. Their molecular identity was refined in 2005 with the revelation of a unique transcriptional signature and the identification of BCL-6 as their master transcription factor. In 2009, the RING-type ubiquitin ligase Roquin was implicated in restraining TFH responses, and more recently, the transcription factors Tox and Tox2 were identified as a pioneer factor cooperating with BCL-6.

More recently, the transcription factors Tox and Tox2 has been identified as a pioneer transcription factor that cooperates with Bcl6 to establish and maintain TFH lineage identity (71). Collectively, these findings position TFH cells as a central conduit between antigen-specific T cell priming and the generation of high-affinity, class-switched antibody responses by B cells, reinforcing the principle that adaptive immunity hinges upon the precise orchestration of T–B lymphocyte synergy (**Figure 3**).

**Figure 4:**
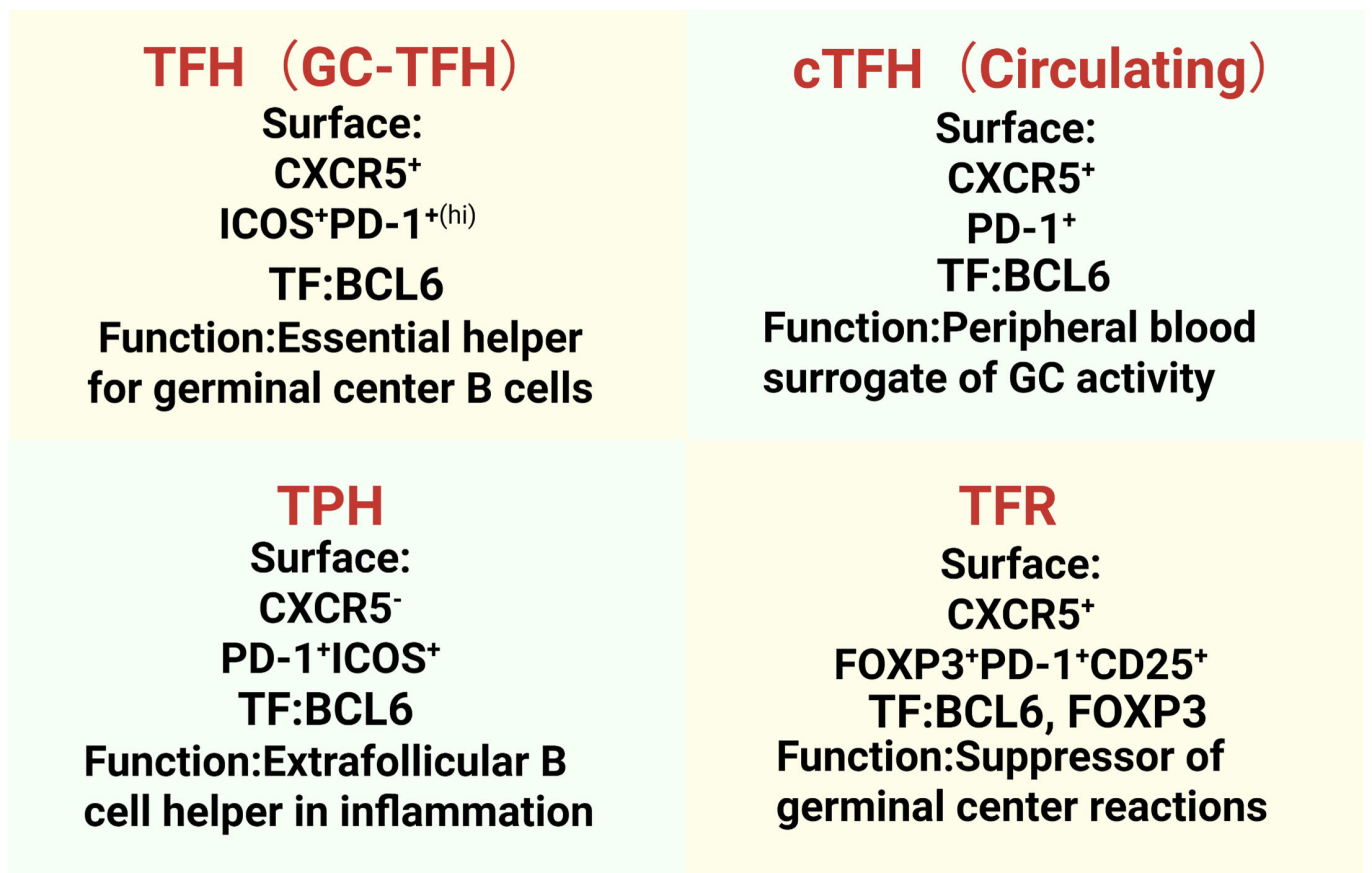
Functional classification and defining markers of TFH-cell subsets. The TFH-cell family comprises four major subsets with distinct roles in antibody responses. Germinal center TFH cells (GC-TFH) are defined by high expression of CXCR5, ICOS, and PD-1, and depend on the transcription factor BCL-6. Circulating TFH cells (cTFH) share CXCR5 and PD-1 expression and serve as a peripheral biomarker. Peripheral helper T cells (TPH) lack CXCR5 but express PD-1 and ICOS, enabling B cell help at extrafollicular inflammatory sites. Follicular regulatory T cells (TFR) co-express the follicular homing marker CXCR5 with the regulatory master transcription factor FOXP3, alongside PD-1 and CD25, and function to suppress excessive immune responses.

**Figure 5:**
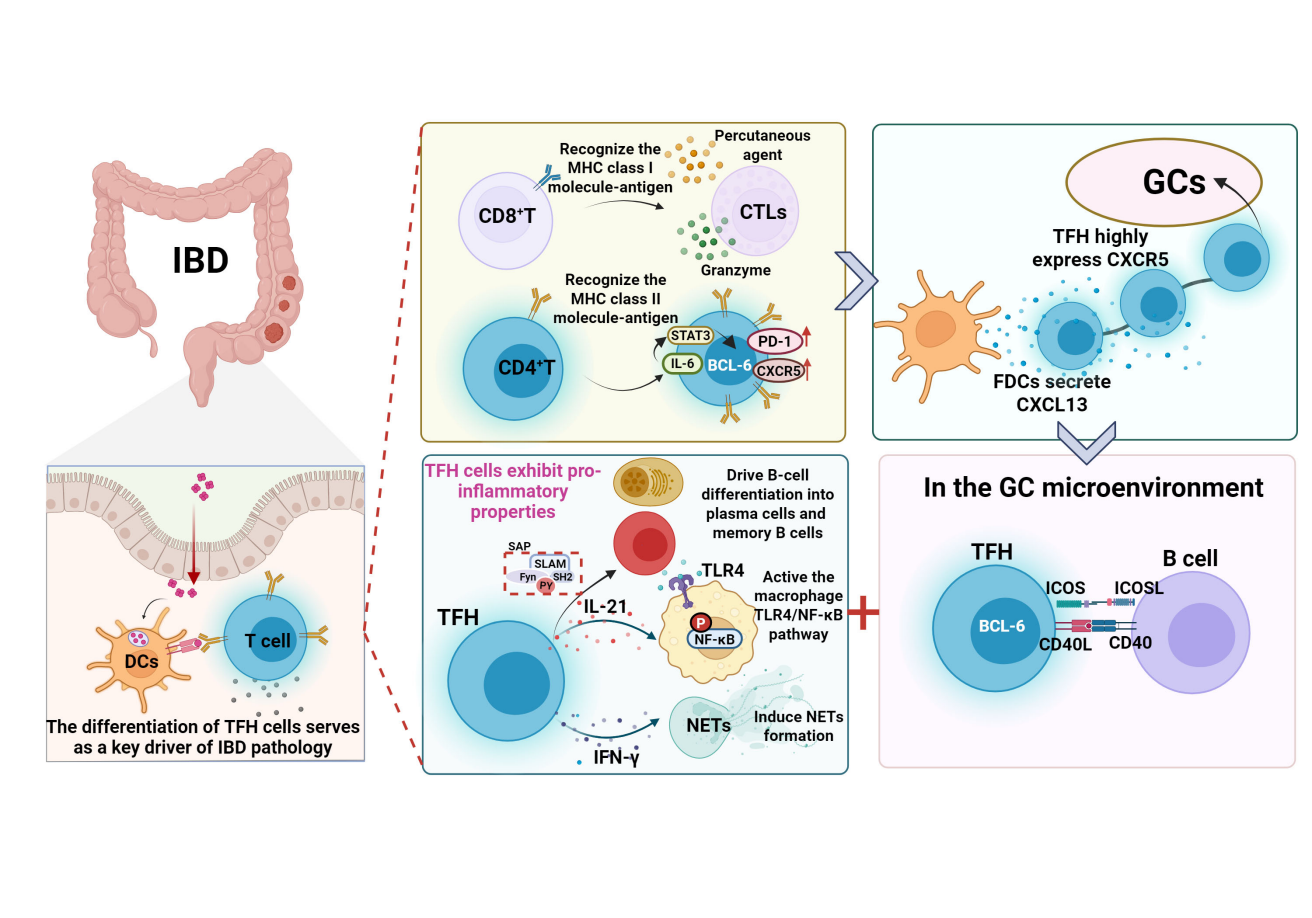
Adaptive immune activation initiates TFH differentiation and pro-inflammatory functions. Activated CD8^+^ T cells differentiate into CTLs and induce target cell apoptosis. CD4^+^ T cells differentiate into subsets including TFH cells, which are core regulators of the humoral immune response. Following dual-signal stimulation, naive CD4^+^ T cells upregulate BCL-6 and acquire the CXCR5^high^PD-1^high^ phenotype. Guided by the CXCR5-CXCL13 axis, TFH cells migrate to germinal centers, where via ICOS/ICOSL, CD40L-CD40, and IL-21, they drive B-cell differentiation. TFH cells also secrete IL-21 and IFN-γ, which can trigger inflammatory cascades, and their abnormal activation is associated with autoimmune diseases.

T-cell activation serves not only as the initiating step in the immune response but also as a key hub of the regulatory network. At the level of cellular immunity, activated CD8^+^ T cells specifically recognize the MHC class I molecule-antigen peptide complex on the surface of target cells through TCRs, differentiate into cytotoxic T lymphocytes (CTLs), and induce target cell apoptosis through the perforin/granzyme system and the Fas/FasL signaling pathway (72, 73). Simultaneously, CD4^+^ T cells recognize the MHC class II molecule-antigen peptide complex on the surface of antigen-presenting cells (APCs) and differentiate into functionally diverse T helper cell subsets. One of these subsets—TFH cells—are the core regulators of the humoral immune response due to their unique localization within GCs and their ability to support B cell functions (74–76). The TFH system primarily includes helper subsets (TFH, TPH, cTFH) and a suppressive subset (TFR), which together regulate the antibody response (Detailed classification shown in **Figure 4**). This functional heterogeneity is reflected in distinct TFH-lineage subsets defined by surface markers and transcriptional programs (**Figure 5**). Classical germinal center TFH cells (hereafter TFH) are identified as CXCR5^+^ICOS^+^PD-1^+^BCL-6^+^ and are essential for providing help to B cells (77, 78). Circulating TFH cells (cTFH; CD4^+^CXCR5^+^PD-1^+^) represent their peripheral counterparts and may serve as a accessible biomarker for systemic TFH activity (79). Peripheral helper T cells (TPH; CXCR5⁻PD-1^+^ICOS^+^) provide B cell help in extrafollicular regions, potentially contributing to early or ectopic antibody responses (80, 81). Crucially, the follicular regulatory T cell subset (TFR; CXCR5^+^FOXP3^+^PD-1^+^) functions as a key suppressive counterpart within follicles (82). In the context of IBD, an imbalance within this system is often observed. Notably, TFR cells, which are critical for constraining excessive germinal center reactions and autoantibody production, are frequently impaired in function or reduced in frequency within inflamed intestinal tissues. This loss of regulatory restraint may directly contribute to the pathological expansion and sustained activity of pro-inflammatory TFH cells, thereby exacerbating chronic inflammation (83).

A typical feature of TFH cells is their high expression of CXCR5 (84). TFH cell differentiation follows a multi-stage, dynamically regulated process. After receiving dual-signal stimulation—TCR-MHC II/antigen peptide presented by APCs—naive CD4^+^ T cells initiate the differentiation program primarily via IL-6-STAT3 signaling, upregulate the expression of the key transcription factor BCL-6, transition through the precursor TFH (pre-TFH) stage and finally acquire the CXCR5^high^PD-1^high^ effector phenotype (18). Importantly, follicular dendritic cells (FDCs) form a chemotactic gradient by secreting CXCL13, guiding TFH cell migration to GC regions through the CXCR5-CXCL13 axis (16, 85, 86). In the GC microenvironment, TFH cells first establish stable contacts with B cells through the inducible T-cell co-stimulator ICOS/ICOSL signaling pathway. Subsequently, through CD40L-CD40 co-stimulation and the simultaneous secretion of IL-21, they drive B-cell differentiation into plasma cells and memory B cells. This process also involves the fine regulation of intercellular synapse formation by the SLAM-associated protein (SAP) –signaling lymphocytic activation molecule (SLAM) interaction network (87–90). Sustained BCL-6 expression maintains the functional maturity of TFH cells by enforcing their transcriptional identity (84).

Meanwhile, mechanistic studies have shown that the immune regulatory function of TFH cells is bidirectional. IL-21 and interferon-gamma (IFN-γ) secreted by TFH cells can not only enhance the intensity of the GC response but also trigger local inflammatory cascades by activating the macrophage TLR4/NF-κB pathway and inducing the formation of neutrophil extracellular traps (NETs) (91). This pro-inflammatory property makes TFH cells an important hub connecting adaptive and innate immunity. Moreover, numerous studies have confirmed that the abnormal activation of TFH cells is associated with a variety of autoimmune diseases, such as rheumatoid arthritis (84, 92). DCs may be significant contributors to intestinal immune homeostasis imbalance through their regulatory effect on the TFH cell differentiation microenvironment. The interaction between the two cell types serves as a key driver of IBD pathology (15, 93) (**Figure 5**).

## 4 The interaction between DCs and TFH cells drives the development of IBD

### 4.1 DCs promote TFH cell differentiation

#### 4.1.1 DCs provide co-stimulatory signals to activate transcription factors and trigger TFH cell differentiation

In IBD pathology, DCs engage in dynamic interactions with TFH cells through a network of co-stimulatory molecules, serving as the core mechanism driving abnormal intestinal immune responses. The binding of B7 family molecules (CD80/CD86) on the surface of DCs, particularly on cDC1 and cDC2 subsets (**Table 1**), to CD28 on naive CD4^+^ T cells not only enhances the phosphorylation of ITAMs within the TCR/CD3 complex through the activation of Lck/Fyn kinases but also stabilizes the binding of ZAP-70 kinase to the CD3ζ chain by inducing conformational changes at the Y315/Y319 sites of ZAP-70 kinase, thereby significantly amplifying the strength of TCR signals (94–97). This synergy lowers the response threshold of T cells to antigens to a pathological level, laying a molecular foundation for the dysregulated differentiation of TFH cells. Notably, OX40L derived from cDC2 cells (**Table 1**) interacts with the OX40 receptor, leading to its trimerization and the subsequent recruitment of TRAF2/3/5 adaptor proteins (98). On the one hand, this results in the activation of the canonical NF-κB pathway through the receptor-interacting protein 1 (RIP1)/inhibitor of nuclear factor kappa-B kinase (IKKα/β/γ) complex, leading to IκBα degradation and the nuclear translocation of reticuloendotheliosis viral oncogene homolog A (RelA)/nuclear factor kappa-B subunit 1 (p50). This mechanism can maintain TFH cell survival even in the absence of antigen stimulation (98–101). On the other hand, the non-canonical NF-κB2 pathway is activated through the CARD-containing MAGUK protein 1 (CARMA1)/B-cell lymphoma/leukemia 10 (BCL-10)/mucosa-associated lymphoid tissue lymphoma translocation protein 1 (MALT1)/protein kinase C theta (PKCθ) complex, inducing the formation of RelB/p52 heterodimers, and, consequently, prolonging NF-κB activity (102). This antigen-independent and sustained signal transduction may be a key trigger for the abnormal activation of TFH cells in IBD. Crucially, the OX40/OX40L axis exhibits unique bidirectional regulatory properties in the GC microenvironment. TFH cells promote IL-21 secretion by receiving B-cell-derived OX40L signals through OX40, while B cells achieve clonal expansion by activating the PI3K-AKT pathway through reverse signaling, forming a positive feedback loop (100, 103, 104). Tahiliani et al. (103) reported that TFH cells in OX40-deficient mice cannot support GC formation, and abnormal GC reactions in the intestinal mucosa are one of the pathological features of IBD. These GC reactions are a hallmark of CD and are also observed in a subset of UC patients, highlighting a shared but variably expressed pathological node. It is noteworthy that OX40/OX40L signaling is context-dependent in immune regulation (105). Under certain experimental conditions, it has also been reported to potentially contribute to the generation of regulatory cells (106), suggesting that its net effect in IBD may be more complex and is precisely regulated by the local microenvironment.

**Figure 6:**
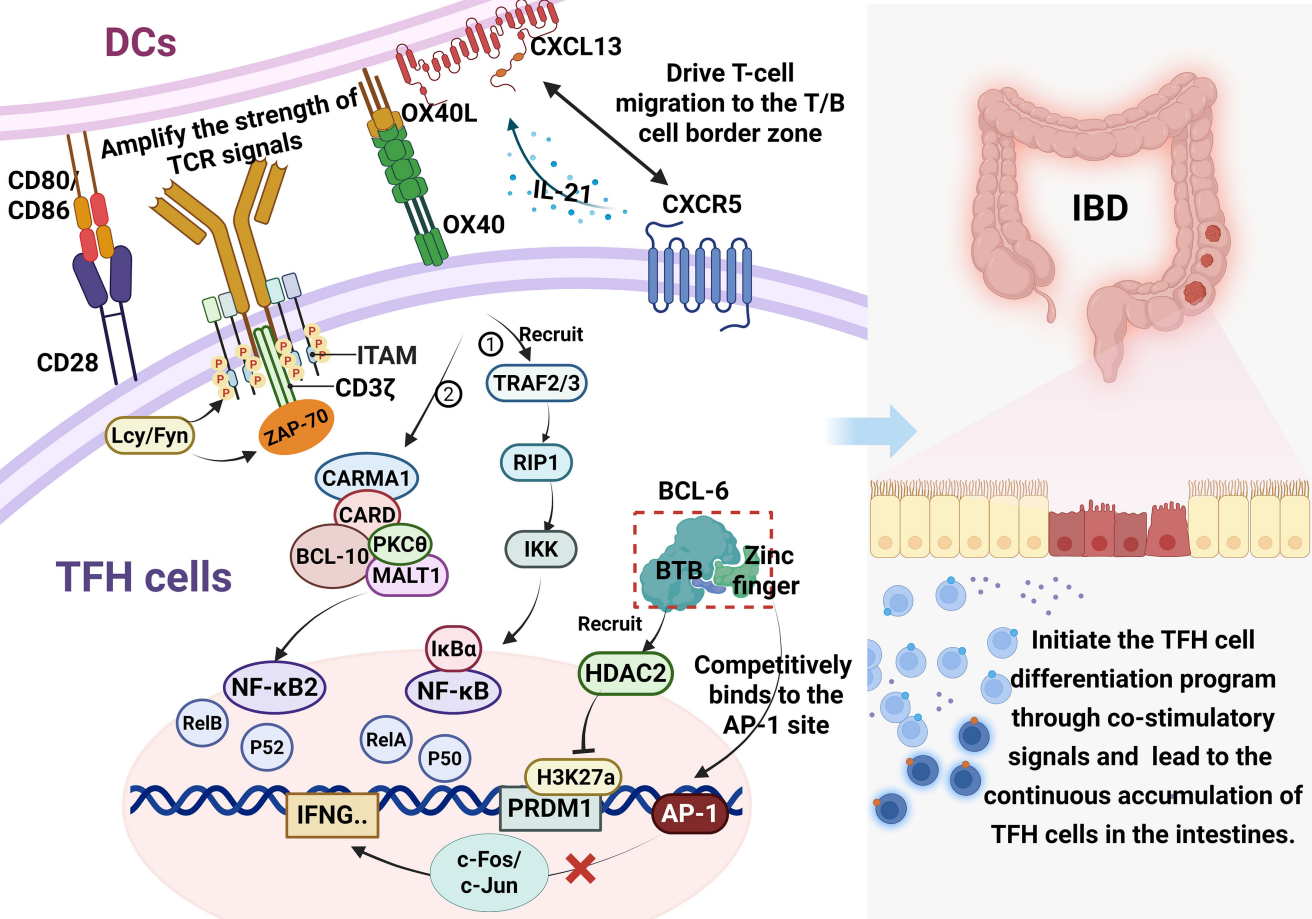
DCs provide co-stimulatory signals to activate transcription factors and trigger TFH cell differentiation. The binding of DC-derived B7 molecules (CD80/CD86) to CD28 on naive CD4^+^ T cells synergistically amplifies TCR signals. Concurrently, the OX40L-OX40 interaction activates both canonical and non-canonical NF-κB pathways, maintaining TFH cell survival and promoting antigen-independent activation. Furthermore, DC-secreted CXCL13 and the BCL-6 transcriptional network coordinate to stabilize CXCR5 expression and enforce TFH lineage commitment. IL-21 from mature TFH cells transactivates CXCL13 in DCs, forming an autocrine amplification loop that may lead to pathological TFH accumulation in IBD.

The regulation of TFH cell differentiation by DCs is also reflected in the precise coordination between the CXCL13-CXCR5 chemokine axis and the BCL-6 transcription network. Although activated CD4^+^ T cells can transiently express CXCR5, the stable expression of this receptor requires DCs to continuously secrete CXCL13 to drive T-cell migration to the T/B cell border zone (91, 107, 108). In this process, BCL-6 relieves the Blimp-1-mediated inhibition of TFH cell differentiation through dual mechanisms. On the one hand, its broad-complex, tramtrack, and bric-à-brac (BTB) domain recruits histone deacetylase 2 (HDAC2), which reduces the level of histone H3K27ac modification at the *PRDM1* promoter region; on the other hand, it competitively binds to the AP-1 site through its zinc finger domain, blocking the c-Fos/c-Jun complex-mediated transcriptional activation of Th1-polarization genes, such as *IFNG* (109, 110). IL-21 secreted by mature TFH cells can transactivate CXCL13 expression in DCs, forming an autocrine amplification loop. This abnormal chemokine-transcription factor network may lead to the continuous accumulation of TFH cells in the intestines of patients with IBD (111). The above-described mechanisms reveal that DCs not only initiate the TFH cell differentiation program through co-stimulatory signals but also maintain the pathological activity of TFH cells by establishing a distinctive immune microenvironment that provides a structural basis for subsequent DCs to secrete cytokines such as IL-12 and TGF-β, thus further modulating TFH cell functions (**Figure 6**).

#### 4.1.2 DCs initiate signal transduction and promote TFH cell differentiation by secreting cytokines

**Figure 7:**
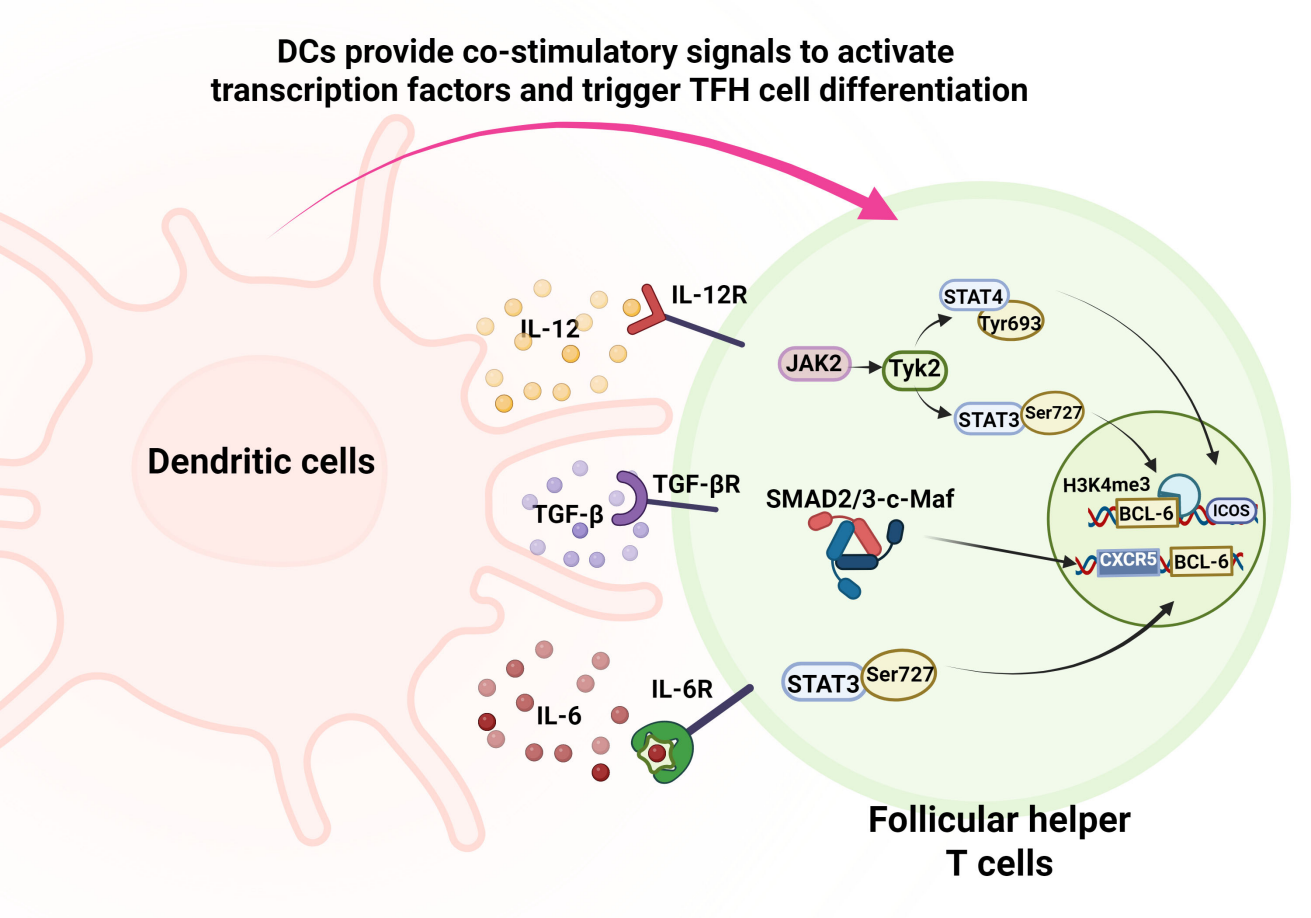
DCs initiate signal transduction and promote TFH cell differentiation by secreting cytokines. DC-derived IL-12 acts via the IL-12Rβ2-JAK2/TYK2-STAT4 axis to activate transcription of BCL-6 and ICOS, while STAT3 Ser727 phosphorylation enhances H3K4me3 modification at the BCL-6 promoter. TGF-β upregulates CXCR5 expression via the SMAD2/3-c-Maf complex, guiding TFH cell migration. Additionally, IL-6 transiently activates STAT3 to induce BCL-6 expression, further reinforcing the TFH differentiation program.

Once DCs have initiated TFH cell differentiation through co-stimulatory signals, they further secrete a range of cytokines that finely regulate the TFH cell differentiation program, constituting a key link in the progression of IBD. Following the initial activation of T cells induced by the B7-CD28 signal on the DC surface, IL-12 derived from cDC1s (**Table 1**) plays a key regulatory role through the IL-12Rβ2-JAK2/TYK2-STAT4 signaling axis. Specifically, the phosphorylation of STAT4 at Tyr693 induces its homodimerization and subsequent translocation to the nucleus, where it directly activates the transcription of BCL-6- and ICOS-encoding genes. Concurrently, STAT3 Ser727 phosphorylation enhances H3K4me3 histone modification in the BCL6 promoter region, thus establishing the epigenetic characteristics of pro-inflammatory TFH cells (112–117). Meanwhile, TGF-β, which is highly expressed by cDC2s and Mo-DCs in the IBD inflammatory microenvironment, directly binds to the CXCR5 promoter through the SMAD2/3-c-Maf complex, thereby upregulating its expression, and promotes TFH cell migration to the GC in a BCL-6-independent manner (118, 119). Human single-cell transcriptome analysis reveals that single-cell transcriptome analysis showed that the proportion of c-Maf^+^ TFH cells in the mesenteric lymph nodes of IBD patients is significantly increased relative to controls, and the CXCR5 expression level in these cells is positively correlated with disease activity index scores (120–122) This suggests that the TGF-β/SMAD pathway exacerbates local intestinal immune responses by modulating the tissue-specific localization of TFH cells. Future studies are needed to delineate whether this c-Maf^+^ TFH signature is equally prominent in CD and UC. In addition, IL-6 secreted by DCs is considered to be an important cytokine driving TFH cell differentiation. Studies in *Il6⁻/⁻ mice* show IL-6 exerts its effects by transiently activating STAT3, which induces BCL-6 expression in CD4^+^ T cells. IBD-related studies have demonstrated that IL-6 deficiency can lead to impaired early-stage TFH cell differentiation, while elevated BCL-6 expression can directly promote CXCR5 upregulation. These findings emphasize the importance of the IL-6/STAT3/BCL-6 axis in the abnormal differentiation of TFH cells in the context of intestinal inflammation in IBD (123) (**Figure 7**).

Notably, DC subsets exhibit differential capacities to drive TFH differentiation. cDC1 cells, through robust IL-12 secretion, preferentially promote TFH subsets with a Th1-like phenotype (sometimes termed TFH1), which may be more relevant in CD (124–126). In contrast, cDC2 cells, via OX40L and TGF-β, support classical TFH differentiation and are often enriched in UC mucosa (127). Mo-DCs, abundant in inflamed tissues, sustain TFH activity through IL-6 and IL-23 (128). Meanwhile, mregDCs may indirectly modulate TFH responses by fostering TFR cell development (129), highlighting the intricate subset-specific crosstalk within the DC–TFH axis.

In summary, DCs precisely regulate multiple signal transduction pathways (JAK-STAT, SMAD) through the secretion of cytokines such as IL-12, TGF-β, and IL-6, synergistically promoting the activation of key transcription factors (BCL-6, c-Maf) and the expression of TFH cell signature molecules (CXCR5), ultimately driving the differentiation and function of TFH cells. This has significant pathological significance for the persistent chronic inflammation and abnormal antibody responses observed in IBD.

### 4.2 TFH cells mediate the recruitment of mature DCs and synergistically drive the formation of DC/T-cell clusters, thus promoting IBD pathology

Furthermore, the abnormal accumulation of mature DCs mediated by TFH cells at IBD lesion sites and the formation of highly ordered “DC/CD4^+^ T-cell clusters” in the T cell zone (15) are also crucial in promoting IBD pathology. Such organized clusters are particularly characteristic of the transmural inflammation seen in CD, whereas in UC, immune aggregates may be more superficially localized within the mucosa, which may partially explain the differential responses of the two subtypes to therapies targeting lymphocyte recruitment. Murine experiments show that, classical GC-TFH cells (**Figure 5**) within the cluster exhibit high expression of lymphotoxin-alpha (LTα). Stimulated by microbial products (such as lipoteichoic acid from Gram-positive bacteria), TFH cells can express both LTα and LTβ. These molecules assemble on the cell surface to form the membrane-bound heterotrimeric ligand LTα1β2. The LTα1β2 trimer binds to the lymphotoxin beta receptor (LTβR) on DCs and stromal cells. This binding triggers the downstream signaling cascade: TRAF2 and TRAF3 recruit cIAP1/2 to the LTβR complex, leading to K63-linked ubiquitination of TRAF3 and its subsequent proteasomal degradation. Since TRAF3 constitutively inhibits NIK, its degradation allows for the stabilization and accumulation of NIK (15, 130, 131). which then activates the non-canonical NF-κB signaling pathway and initiates the transcription of specific chemokines, such as CXCL13, CCL19, and CCL21 (132, 133). These chemokines further recruit additional CXCR5^+^ lymphocytes (including TFH cells) and CCR7^+^ mature DCs, including the cDC1 and cDC2 subsets (**Table 1**), to the IBD lesions (134), thus exacerbating DC accumulation and promoting the expansion of DC/T-cell clusters.

T cells within the expanded DC/T-cell clusters receive significantly enhanced and prolonged antigenic signals from DCs, leading to excessive activation of CD4^+^ T cells and their abnormal differentiation into various pro-inflammatory effector T-cell subsets, including Th1 and Th17 cells (12), In the murine DSS model, this process is reproduced, human single-cell data similarly show expanded Th1/Th17 modules in active IBD, this is often accompanied by the inhibition of the function or differentiation of Tregs (see below). These differentiated effector T cells migrate to the intestinal lamina propria, exerting pro-inflammatory effects and directly driving IBD pathology. Notably, the differentiation of Th1 cells and their effector mechanisms are particularly critical. IL-12, secreted by DCs, binds to IL-12R and activates JAK2/TYK2-STAT4 signaling, which, in turn, induces the expression of T-box transcription factor (T-bet) (135). Human genetic studies complement these murine observations: T-bet directly drives the Th1 cell differentiation program and promotes massive IFN-γ production. IFN-γ further strengthens T-bet expression through the STAT1 phosphorylation (136), forming a positive feedback loop. Genetic studies of IBD patients (137) (involving SNP rs1551398/rs1551399, which affects T-bet binding) and functional studies (138) (involving the alleviation of IBD through IFN-γ deficiency in an animal model) both support the importance of Th1 cell responses. Activated Th1 cells secrete cytokines, such as IFN-γ and tumor necrosis factor-alpha (TNF-α), which stimulate macrophages/neutrophils, upregulate the expression of epithelial adhesion molecules (e.g., MAdCAM-1), promote immune cell infiltration, and directly or indirectly induce intestinal epithelial cell apoptosis and dysfunction, thereby disrupting the intestinal barrier (136, 138).

**Figure 8:**
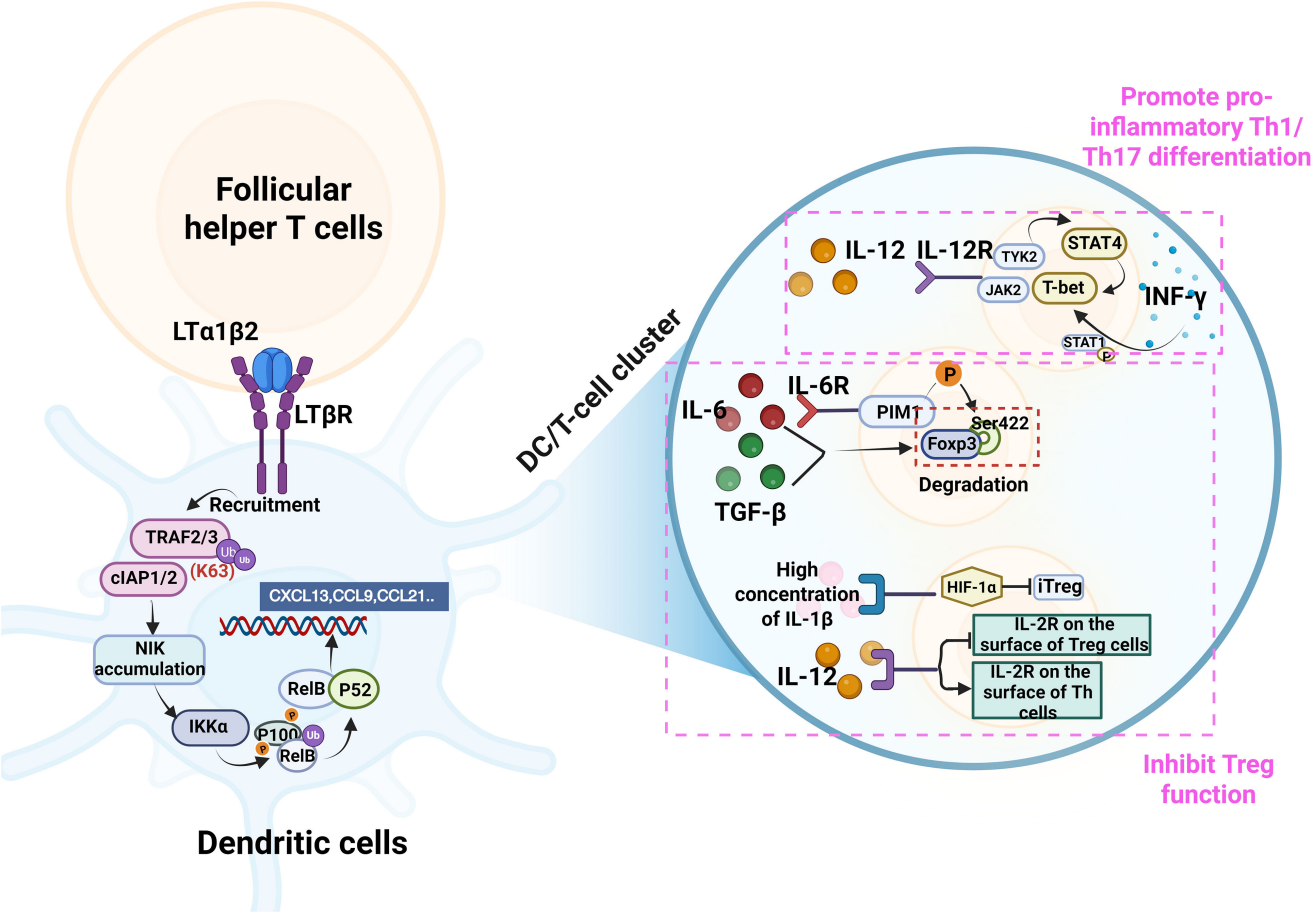
TFH cells mediate the recruitment of mature DCs and synergistically drive the formation of DC/T-cell clusters, thus promoting IBD pathology. TFH-derived membrane-bound LTα1β2 binds to LTβR on DCs, triggering a non-canonical NF-κB signaling cascade (via TRAF3 degradation/NIK stabilization) that induces chemokine (CXCL13, CCL19, CCL21) production. This recruits more immune cells, expanding the clusters. Within clusters, enhanced DC signals promote pro-inflammatory Th1/Th17 differentiation (e.g., via the IL-12-STAT4-T-bet-IFN-γ axis) while inhibiting Treg function through cytokines (IL-6, TGF-β, IL-12, IL-1β), collectively exacerbating intestinal.

In addition to driving Th1 cell differentiation, the DC/T-cell cluster microenvironment can also disrupt immune tolerance by interfering with Treg differentiation and function, thus participating in the pathogenesis of IBD. Both animal studies and human specimen analyses have shown a reduced frequency of FOXP3^+^ regulatory T cells within active lesions. Key cytokines secreted by DCs, including IL-6, TGF-β, IL-12, and IL-1β, have concentration-dependent regulatory effects on Tregs. IL-6 can inhibit the transcriptional activity and anti-inflammatory function of Tregs by inducing the expression of the kinase proviral integration site for Moloney murine leukemia virus 1 (PIM1), which phosphorylates FOXP3 at Ser422 (139). Although TGF-β is a key inducer of FOXP3 expression, it can synergize with IL-6 to promote FOXP3 protein degradation and inhibit Treg function in the inflammatory environment of IBD (140). Similarly, high concentrations of IL-1β significantly inhibit the differentiation of naive T cells into FOXP3^+^ iTregs as well as the expression of FOXP3 by upregulating hypoxia-inducible factor 1 alpha (HIF-1α) under TCR stimulation. In contrast, low IL-1β concentrations may have a Treg proliferation-inducing effect (141). IL-12 exhibits bidirectional regulation, elevating the expression of IL-2 receptors on effector T cells, while concomitantly downregulating that of IL-2R in Tregs, thereby competitively limiting the critical support of IL-2 for Treg survival and function (142). These mechanisms collectively lead to the inhibition of Treg function and exacerbate the immune imbalance in IBD (**Figure 8**). The relative contribution of these cytokine-mediated disruptions to Treg function may vary between CD and UC, reflecting their distinct inflammatory milieus. In addition, it should be noted that the TFH-DC axis is clearly distinct from the germinal-center–dependent paradigm of the classical TFH-B axis. To visualize this difference, the study provides a systematic side-by-side comparison of the core components of TFH-B versus TFH-DC interactions in **Table 2**, focusing on three dimensions —cytokine networks, co-stimulatory signals, and chemotactic axes —thereby highlighting the unique position of the TFH-DC axis in the progression of IBD (**Table 2**).

**Table 2:** Systematic comparison of TFH-B versus TFH-DC interactions: analyzing the pathogenic significance and unique features of TFH-DC for IBD.

Comparison dimension	TFH-B interactions	TFH-DC interactions	Pathogenic significance and uniqueness in IBD
Key Cytokine Network	IL-21: Drives B cell clonal expansion and differentiation (100, 103, 104).	IL-12/IL-23: Drives TFH function (150, 151); TGF-β: Cooperates with IL-6 to drives pathogenic TFH differentiation in IBD (140); IL-6: Facilitates TFH localization and differentiation via SMAD-c-Maf–driven CXCR5 upregulation (118, 119).	DC-derived IL-12 and TGF-β sequentially supply the survival and differentiation signals that convert naive CD4^+^ T cells into CXCR5^+^ TFH cells, a DC-TFH reciprocity not required for conventional T-B help (112–121).
Co-stimulatory Signals	OX40-OX40L: TFH cells promote IL-21 secretion by receiving B-cell-derived OX40L signals through OX40, while B cells achieve clonal expansion by activating the PI3K-AKT pathway through reverse signaling, forming a positive feedback loop (100, 103, 104)**; ICOS/ICOSL**: In the GC microenvironment, TFH cells first establish stable contacts with B cells (87–90).	CD80/CD86-CD28: Lowers the response threshold of T cells to antigens to a pathological level, laying a molecular foundation for the dysregulated differentiation of TFH cells (64, 65); OX40L-OX40: Maintains TFH cell survival in the absence of antigen stimulation, prolonging NF-κB activity and contributing to chronic intestinal inflammation (98–101).	Compared with the TFH–B-cell axis, the TFH-DC interface employs the same molecular pair to build an antigen-independent circuit: DCs first “lower the response threshold of T cells to antigens to a pathological level” via CD80/CD86-CD28, and then “maintain TFH cell survival in the absence of antigen stimulation, prolonging NF-κB activity” through OX40L-OX40, thereby sustaining chronic intestinal inflammation (94–97, 100, 103, 104).
Chemokine Axis	CXCL13(secreted by DCs)-CXCR5 (on TFH): Guides TFH homing to T/B cell border zone (91, 107, 108).	CXCL13-CXCR5: Recruits TFH and B cells to inflammatory sites, forming pathological structures (91, 107, 108). CCL19/CCL21-CCR7: Recruits CCR7^+^ mature DCs to T-cell zones, fostering cluster formation (132–134).	In the TFH-DC axis, production of CXCL13 is driven by the TFH-derived LTα1β2-LTβR signal, creating a self-reinforcing recruitment hub central to the formation of inflammatory clusters (16, 132, 133).

In summary, the pathogenesis of IBD can be reframed as a breakdown in subset-specific communication: pro-inflammatory DC subsets (e.g., cDC2, Mo-DC; **Table 1**) and TFH-lineage effectors (e.g., GC-TFH; **Figure 4**) engage in a vicious cycle, while regulatory counterparts (e.g., mregDC, TFR) fail to impose restraint, culminating in chronic intestinal inflammation.

## 5 Therapeutic targeting of the DC-TFH cell interactions delays IBD progression

Effective therapeutic strategies are built upon systematic summarizing and parsing of the DCs–TFH interaction mechanism. To this end, this study first systematically compares species-specific findings from animal models against the clinical characteristics of the corresponding diseases, and then uses the attached table to analyse their translational potential, thereby providing solid evidence-based support for the formulation of subsequent clinical intervention strategies (**Table 3**).

**Table 3:** Model-specific findings versus the clinical characteristics of IBD: analyzing translational relevance.

Animal model studies	Clinical characteristics of IBD	Translational relevance
DCs in inflamed gut show high CD80/CD86, OX40L; TFH are CXCR5^+^PD-1^+^BCL-6^+^ (18, 54, 55, 94–97).	Increased c-Maf^+^ TFH cells in mesenteric lymph nodes of IBD patients; correlation with disease activity (120–122).	In colitis models, mucosal DCs up-regulate CD80/CD86 and OX40L and generate CXCR5^+^PD-1^+^BCL-6^+^ TFH cells (18, 54, 55, 94–97); the same pathway is echoed clinically by the selective expansion of c-Maf^+^ TFH cells in mesenteric lymph nodes of IBD patients that tracks with disease activity, confirming that the DC–TFH interactions is a translatable biomarker and therapeutic target (120–122).
IL-12/STAT4/BCL-6 axis drives TFH differentiation (105–110); TGF-β/c-Maf/CXCR5 axis promotes migration (118, 119).	Human single-cell data support roles of IL-12/STAT4 and TGF-β/SMAD pathways in TFH differentiation (112–117).	IL-12/STAT4 and TGF-β/SMAD circuits identified in murine TFH differentiation are echoed by human single-cell data endorsing continued use of IL-12/IL-23 blockade (ustekinumab) and prompting exploration of TGF-β-directed agents, while remaining alert to species-specific receptor expression patterns (112–117).
DSS/TNBS colitis shows TFH expansion, enhanced GC reactions, and DC-TFH-driven tertiary lymphoid structure formation (16).	Tertiary lymphoid structures and CXCL13^+^ DC–TFH clusters observed in intestinal tissues of IBD patients (132, 133).	DSS/TNBS coline models demonstrate that TFH expansion, exaggerated germinal-center reactions and DC-TFH-driven tertiary lymphoid structure formation are the mechanistic core of chronic gut inflammation (16); the identical tertiary lymphoid structure and CXCL13^+^ DC-TFH clusters documented in intestinal biopsies from IBD patients validate these pathways as translatable therapeutic targets (132, 133).
Anti-IL-6R (tocilizumab analogs) and JAK inhibitors suppress TFH differentiation and ameliorate colitis in mice (153, 154).	Clinically used biologics (e.g., ustekinumab, vedolizumab) modulate TFH-associated immune responses in IBD patients (152, 153).	Murine studies demonstrate that anti-IL-6R antibodies and JAK inhibitors blunt TFH differentiation and curtail experimental colitis (153, 154), while the same clinically approved biologics ustekinumab and vedolizumab replicate this suppression of TFH-associated immunity in IBD patients, confirming the axis as an immediately translatable therapeutic target (152, 153).
Mouse models reveal LTα1β2-LTβR axis drives DC-TFH clustering and lymphoid neogenesis (15, 130, 131).	Spatial transcriptomics in human IBD tissues shows CXCL13–CXCR5 co-localization, suggesting similar microenvironmental cues (111).	Mouse studies establish that the LTα1β2-LTβR axis generates CXCL13-CXCR5-dependent DC-TFH clusters and lymphoid neogenesis (15, 130, 131); spatial transcriptomics of human IBD mucosa now reveals the same CXCL13-CXCR5 co-localization, confirming that this microenvironmental cue is evolutionarily conserved and immediately targetable with anti-LTβR or CXCL13/CXCR5 blockers (111).

As previously mentioned, the interaction between DCs and TFH cells plays a central driving role in the occurrence and development of IBD through the establishment of a vicious cycle (DCs drive TFH cell differentiation, while TFH cells promote DC accumulation and DC/T-cell cluster formation). Therefore, disrupting this interaction has emerged as an important therapeutic strategy for IBD. Given the relative stability of DCs as innate immune cells, current therapeutic approaches mostly focus on inhibiting the development and function of TFH cells, thereby indirectly regulating DC-TFH cell interactions. It is noteworthy that the clinical application and evidence base for these therapies often differ between CD and UC. Aminosalicylates, including sulfasalazine and mesalazine, are conventional therapeutic agents primarily used in UC that mainly exert anti-inflammatory effects by inhibiting excessive immune cell activation. *In vivo* studies have shown that these drugs can downregulate the expression of the pro-inflammatory factor TNF-α and upregulate that of the anti-inflammatory factor IL-10, thereby inhibiting immune responses and promoting intestinal mucosal barrier repair (143–145). Crucially, given the important role of TNF-α in the promotion of TFH cell differentiation, aminosalicylates can indirectly influence the development of TFH cells by inhibiting this cytokine (143).

Prednisolone suppresses DC maturation through the GR –NF-κB axis, which in turn downregulates IL-6/IL-21 production, thereby impairing TFH cell differentiation and function. Ultimately, this leads to a reduction in germinal center reactions and autoantibody production. This pathway not only complements the traditional anti-inflammatory mechanism of glucocorticoids at the level of humoral immune suppression but also provides a theoretical basis for combination therapies targeting TFH cell in IBD (146). Furthermore, Cyclosporine A acts by blocking the calcineurin–NFAT axis, resulting in the downregulation of DC-derived IL-6/IL-21 and reduced expression of ICOS/PD-1 on TFH cell. This rapidly suppresses intestinal TFH cell differentiation and germinal center reactions during acute phases of IBD, thereby reducing autoantibody production (147, 148). Similarly, Tacrolimus inhibits the calcineurin–NFAT axis, leading to decreased DC-derived IL-6/IL-21 and reduced CD40L expression on CD4^+^ T cells. This similarly results in rapid suppression of intestinal TFH cell differentiation, germinal center reactions, and autoantibody generation during acute IBD flares (149, 150). Together, these agents provide a rational basis for the treatment of severe IBD refractory to corticosteroid therapy.

Randomized clinical trials and real-world clinical experience alike have demonstrated that ustekinumab —a monoclonal antibody targeting the p40 subunit shared by IL-12 and IL-23 (151), blocks the binding of both cytokines to their receptors and inhibits downstream pro-inflammatory signaling. As IL-23 plays a crucial role in maintaining the function of TFH cells, ustekinumab can also interfere with TFH cell development and homeostasis, thereby alleviating IBD inflammation (152). It is an approved therapy for both moderate-to-severe CD and UC. In the inflammatory microenvironment of IBD, tocilizumab, a monoclonal antibody targeting the IL-6 receptor, suppresses the generation of TFH cells by blocking IL-6 signaling and also maintains BCL-6 expression at low levels (153). While primarily used in other autoimmune diseases, its role in IBD remains investigational. Olamkicept, a soluble gp130-Fc fusion protein that selectively inhibits IL-6 trans-signaling, has shown efficacy and safety in clinical studies for active UC (154); however, excessive inhibition of IL-6 signaling, such as occurs with tocilizumab, can potentially exacerbate IBD (155). The abnormal elevation of TNF-α is a key feature of IBD. Anti-TNF-α monoclonal antibodies, such as infliximab and adalimumab, effectively reduce peripheral blood T-cell activation (manifested as the downregulation of CD25 expression); inhibit the secretion of pro-inflammatory factors such as IFN-γ, IL-13, IL-17A, and TNF; and suppress the proliferation of CD4^+^ and CD8^+^ T cells by neutralizing TNF-α (156, 157). These agents are cornerstone therapies for both CD and UC. Because TNF-α participates in the regulation of TFH cell differentiation, anti-TNF-α therapy can also indirectly affect the TFH cell pool.

Vedolizumab, an anti-integrin drug, prevents lymphocyte homing to the intestinal mucosa via the dual blockade of α4β7 and αEβ7 integrins, thereby inhibiting the initial activation of the intestinal mucosal immune system and limiting the local accumulation of effector T cells, including CD8^+^ T cells (158). The intestinal mucosa is a key site for TFH cell function. These cells express high levels of CXCR5 and migrate to the GC to assist B cells in differentiating into plasma cells and producing antibodies (159). By blocking lymphocyte homing, vedolizumab indirectly interferes with intestinal T-cell activation pathways, including TFH cell-mediated B cell assistance, thereby dampening local immune responses and alleviating IBD (160, 161). It is effective for inducing and maintaining remission in both UC and CD.

The JAK inhibitor upadacitinib—currently approved for moderate-to-severe UC and with emerging evidence in CD—simultaneously inhibits signal transduction mediated by key cytokines (such as IL-6, IL-12, and IL-23), influencing TFH cell development by blocking the JAK-STAT signaling pathway. This effectively reduces intestinal mucosal inflammation and helps control IBD progression (162).

S1P receptor modulators, such as Ozanimod (approved for moderate−to−severe UC and with Phase III evidence in CD) and Etrasimod (approved for UC and demonstrating efficacy in CD trials), reduce the homing of CD11c^+^ activated DCs to the intestinal mucosa while downregulating DC-derived IL-12/IL-23. This indirectly suppresses the TFH cell differentiation axis, leading to reduced infiltration of immune cells in the intestinal mucosa and promoting repair of the mucosal barrier, thereby alleviating clinical symptoms of IBD (163) (**Table 4**).

**Table 4:** Treatment strategy of the DC-TFH cell interactions delays IBD progression.

Classification	Drug	Mechanism	References
Aminosalicylic acids	Sulfasalazine Mesalazine	Upregulation of IL-10 and downregulation of TNF-α indirectly inhibit TFH cell differentiation.	(143–145)
Glucocorticoids	Prednisolone	Inhibition of DCs maturation *via* the GR-NF-κB axis leads to decreased production of IL-6 and IL-21, resulting in suppressed TFH cell differentiation and GC response.	(146)
Immunosuppres-sant	Cyclosporine A	Through blocking the calcineurin-NFAT axis, this leads to reduced production of IL-6 and IL-21 by DCs and downregulation of ICOS/PD-1 on TFH cells, thereby acutely suppressing TFH differentiation and GC responses.	(147, 148)
	Tacrolimus	Through blocking the calcineurin-NFAT axis, this leads to reduced production of IL-6 and IL-21 by DCs and downregulation of CD40L expression, thereby acutely suppressing TFH cell differentiation and GC reactions.	(149, 150)
Biologics	Ustekinumab	Targeting p40 inhibits IL-23, thereby suppressing TFH cell development and homeostasis.	(151, 152)
	Tocilizumab Olamkicept	Blockade of the IL-6R leads to suppressed IL-6 signaling, resulting in low BCL-6 expression and consequently inhibiting the generation of TFH cells.	(153, 154)
	Infliximab Adalimumab	Neutralizing TNF-α leads to reduced CD25 expression and decreased pro-inflammatory cytokines, thereby indirectly inhibiting the differentiation and expansion of TFH cells.	(156, 157)
	Vedolizumab	Through blocking lymphocyte homing via targeting α4β7/αEβ7 integrins, the provision of TFH cell support for GC reactions in the intestinal mucosa is suppressed, thereby alleviating local immune responses.	(160, 161)
JAK inhibitors	Upadacitinib	Through selective JAK1 inhibition, the IL-6/12/23-JAK-STAT signaling pathway is blocked, leading to suppressed TFH cell development.	(162)
S1P receptor modulators	Ozanimod Etrasimod	Inhibition of DCs homing to the intestinal mucosa leads to reduced production of IL-12 and IL-23, thereby indirectly suppressing TFH cell differentiation.	(163)
Chinese herbal medicines or their active ingredients	Sishen Wan	Through inhibiting the BCL-6/Blimp-1 pathway, thereby suppressing TFH cell differentiation.	(164)
	Paeoniflorin	Inhibiting PI3K-AKT-dependent DC maturation and DC–IL-6-driven TFH differentiation.	(165)
	Artesunate	Blocking JAK2-STAT3-BCL-6 signaling to suppress TFH differentiation.	(166)
	Xiehuo Xiaoying Decoction	Suppression of TFH expansion and promotion of Tfr proliferation to rebalance TFH/Tfr and slow autoimmune progression.	(167)
	Ginsenoside	Blocking TLR recognition, inducing SOCS, inhibiting PI3K-AKT/STAT, expanding Treg, and suppressing TFH function	(168)
	Luteolin	Inhibiting p-JAK1/p-STAT1-mediated TFH differentiation.	(171)

In addition, growing evidence suggests that traditional Chinese medicine and its active ingredients can also affect the progression of IBD through the regulation of TFH cell development and DC-TFH cell interactions. However, it should be noted that many preclinical studies utilize UC-like animal models [e.g., Dextran sulfate sodium (DSS)-induced colitis], and their findings may require further validation in CD-relevant models. For instance, the formula Sishen Wan alleviates colitis symptoms in mice by inhibiting the Bcl-6/Blimp-1 pathway and suppressing TFH cell differentiation (164). Paeoniflorin, the main active ingredient in Paeonia lactiflora, has been shown in a 3% DSS-induced UC mouse model to inhibit PI3K-AKT-dependent dendritic cell maturation, impair DC-derived IL-6-mediated TFH cell differentiation, and subsequently reduce IL-21 production and downstream inflammatory cascades, thereby ameliorating experimental colitis. This provides direct evidence supporting the “TCM monomer-DC-TFH interaction” paradigm for UC treatment (165).

Building on this successful paradigm, artesunate alleviates autoantibody responses in lupus-prone mice by blocking TFH differentiation via a JAK2-STAT3-Bcl-6-dependent mechanism (166); Xiehuo Xiaoying Decoction has been demonstrated to modulate the TFH/Tfr cell balance by suppressing TFH cell expansion and promoting Tfr cell proliferation, thereby delaying the progression of autoimmune-related diseases (167); ginsenoside can block recognition by TLRs and induce the expression of suppressor of cytokine signaling (SOCS), inhibit the PI3K-AKT and STAT signaling pathways, increase the Treg proportion, and inhibit TFH cell function (168–170); and luteolin has been shown to inhibit p-STAT1 and p-JAK1 expression related to TFH cell differentiation, block NF-κB signal transduction, and significantly reduce the levels of cyclooxygenase-2 (COX-2), inducible nitric oxide synthase (iNOS), IL-8, and nitric oxide (NO) in colon tissue, thereby improving IBD-like symptoms (171). These studies collectively provide valuable insights for developing novel therapeutic strategies for IBD (**Table 4**).

## 6 Summary and prospects

IBD is characterized by a high incidence and complex pathogenesis. Recent research has revealed that the immunological interaction between DCs and TFH cells plays a key role in IBD occurrence and development. Nevertheless, significant gaps in current knowledge remain. First, direct spatial evidence of DC-TFH interactions in human IBD tissues is still limited. Second, the dynamics of their molecular interplay across different disease stages and subtypes are not fully elucidated. Finally, the potential long-term impact of targeting this axis on systemic immune surveillance warrants careful evaluation. While this axis appears central to both CD and UC, its specific cellular and molecular dynamics may be shaped by the distinct inflammatory milieus of each subtype. Building on emerging insights into how DC-TFH cell interactions drive IBD pathology, in this review, we summarized current clinical drug strategies aimed at targeting this axis to mitigate disease symptoms. However, the unavoidable side effects associated with these therapies limit their application prospects. Accordingly, further exploration of the unique advantages of TCM-notably, its multi-component, multi-target, and multi-pathway modulation of immune networks-holds considerable therapeutic promise for IBD. This approach may be particularly suited to address the heterogeneous immune dysregulation seen across IBD subtypes.

To address these knowledge gaps, future investigations should employ high-resolution technologies—such as single-cell RNA sequencing and spatial transcriptomics—to map the subset-specific interaction landscape between DCs and TFH cells in IBD tissues. Elucidating how distinct DC subsets (e.g., cDC1, cDC2, Mo DC, mregDC) communicate with TFH-lineage populations (including TFH, TFR, and cTFH) in a spatially organized manner will not only refine our understanding of disease mechanisms but also pave the way for subtype tailored therapeutic strategies in CD versus UC.

This high-resolution, subset-focused paradigm should also guide the investigation of traditional therapeutic agents, such as TCM. Although studies have shown that TCM can alleviate IBD symptoms by inhibiting the expression of key cytokines and transcription factors involved in TFH cell differentiation, thereby indirectly interfering with DC/TFH cell interactions. Nevertheless, direct experimental evidence confirming that TCM components act on the DC/TFH cell interface remains limited. Future research should prioritize integrating high-resolution technological platforms, such as single-cell RNA sequencing, multiplexed immunofluorescence *in situ* hybridization, and spatial transcriptomics, to systematically dissect the molecular mechanisms through which TCM regulates direct immune cell interactions, particularly between DCs and TFH cells. Crucially, these investigations should be conducted with explicit attention to distinguishing the profiles and interplay of these cells in CD versus UC, to uncover subtype-specific therapeutic targets. This will provide crucial directions for elucidating the immunopharmacological foundations of TCM in the treatment of IBD.

## Glossary

IBD: inflammatory bowel disease
DCs: dendritic cells
TFH: T follicular helper
LTα1β2: lymphotoxin alpha 1 beta 2
CD: Crohn’s disease
UC: ulcerative colitis
CXCR5: C-X-C motif chemokine receptor 5, GCs, germinal centers
CXCL13: C-X-C motif chemokine 13
ICOSL: inducible co-stimulator ligand
IL-21: interleukin-21
Th1: T helper 1
cDCs: conventional dendritic cells
pDCs: plasmacytoid dendritic cells
PAMPs: pathogen-associated molecular patterns
PRRs: pattern recognition receptors
TLRs: Toll-like receptors
CLRs: C-type lectin receptors
SRs: scavenger receptors
TGF-β: transforming growth factor-beta
JAK1: Janus kinase 1
TYK2: tyrosine kinase 2
STAT1: signal transducer and activator of transcription 1
PI3K: phosphatidylinositol 3-kinase
PI3K: phosphatidylinositol 3-kinase
TGF-βRII: TGF-β
TGF-βRI: TGF-β
FKBP1A: FK506-binding protein 1A
SMAD: small mothers against decapentaplegic
R-SMADs: receptor-regulated small mothers against decapentaplegic (SMAD) proteins
Tregs: regulatory T cells
LPS: lipopolysaccharide
IRAK1/4: interleukin-1 receptor-associated kinase 1/4
MyD88: myeloid differentiation factor 88
TRAF6: tumor necrosis factor receptor-associated factor 6
TAK1: transforming growth factor β-activated kinase 1
NF-κB: nuclear factor-kappa B
MAPK: mitogen-activated protein kinase
Lck: lymphocyte-specific tyrosine kinase
ITAMs: immunoreceptor tyrosine-based activation motifs
ZAP-70: ζ-chain-associated protein kinase 70
PI3K: phosphatidylinositol 3-kinase
NFAT: nuclear factor of activated T-cells
AP-1: activator protein 1
CTLs: cytotoxic T lymphocytes
APCs: antigen-presenting cells
pre-TFH: precursor TFH
FDCs: follicular dendritic cells
SAP: SLAM-associated protein
SLAM: signaling lymphocytic activation molecule
IFN-γ: interferon-gamma
NETs: neutrophil extracellular traps
RIP1: receptor-interacting protein 1
IKK: inhibitor of nuclear factor kappa-B kinase
RelA: reticuloendotheliosis viral oncogene homolog A
CARMA1: CARD-containing MAGUK protein 1
BCL-10: B-cell lymphoma/leukemia 10
MALT1: mucosa-associated lymphoid tissue lymphoma translocation protein 1
PKCθ: protein kinase C theta
BTB: bric-à-brac
HDAC2: histone deacetylase 2
LTα: lymphotoxin-alpha
LTβR: lymphotoxin beta receptor
T-bet: T-box transcription factor
TNF-α: tumor necrosis factor-alpha
PIM1: proviral integration site for Moloney murine leukemia virus 1
HIF-1α: hypoxia-inducible factor 1 alpha
SOCS: suppressor of cytokine signaling
COX-2: cyclooxygenase-2
iNOS: inducible nitric oxide synthase
NO: nitric oxide
DSS: Dextran sulfate sodium.
